# Encounters in Three Dimensions: How Nuclear Topology Shapes Genome Integrity

**DOI:** 10.3389/fgene.2021.746380

**Published:** 2021-10-21

**Authors:** Robin Sebastian, Mirit I. Aladjem, Philipp Oberdoerffer

**Affiliations:** ^1^ Developmental Therapeutics Branch, Center for Cancer Research, National Cancer Institute, NIH, Bethesda, MD, United States; ^2^ Division of Cancer Biology, National Cancer Institute, NIH, Rockville, MD, United States

**Keywords:** genome integrity, nuclear organization, replication stress, Topologically Associated Domain, chromatin, DNA double-strand break repair, phase separation

## Abstract

Almost 25 years ago, the phosphorylation of a chromatin component, histone H2AX, was discovered as an integral part of the DNA damage response in eukaryotes. Much has been learned since then about the control of DNA repair in the context of chromatin. Recent technical and computational advances in imaging, biophysics and deep sequencing have led to unprecedented insight into nuclear organization, highlighting the impact of three-dimensional (3D) chromatin structure and nuclear topology on DNA repair. In this review, we will describe how DNA repair processes have adjusted to and in many cases adopted these organizational features to ensure accurate lesion repair. We focus on new findings that highlight the importance of chromatin context, topologically associated domains, phase separation and DNA break mobility for the establishment of repair-conducive nuclear environments. Finally, we address the consequences of aberrant 3D genome maintenance for genome instability and disease.

## Introduction

Eukaryotic genomes are exposed to numerous sources of DNA damage, of which DNA double-strand breaks (DSBs) are arguably the most deleterious. DSBs can arise from exposure to genotoxic agents, many of which are used in cancer therapy, but they can also be the result of endogenous processes such as oxidative metabolism and DNA replication. Aberrant repair of DSBs can cause chromosomal translocations, genomic duplications or deletions, as well as DNA mutations, all of which may result in defective cell function, cell death or malignant transformation.

Three main pathways exist to repair DSBs in mammalian cells: i) non-homologous end-joining (NHEJ), a fast but error-prone re-ligation of broken DNA ends; ii) microhomology-mediated end joining (MMEJ, also known as alternative end joining or alt-EJ), a process that relies on moderate DNA end resection and frequently results in small insertions or deletions (indels); and iii) homologous recombination (HR), which is a templated process and therefore considered error-free, but generally restricted to S/G2 phases of the cell cycle (Jasin and Rothstein, 2013; Chang et al., 2017). While both error-free and potentially error-prone repair pathways play essential roles in genome maintenance, inappropriate repair pathway choice can have detrimental consequences for genome integrity (Scully et al., 2019). The latter can be the result of DNA repair factor mutations, as often observed in cancer cells (e.g., breast and ovarian cancers with defective BRCA genes), but further depends on a more complex set of temporal and local factors, most notably cell cycle phase and the DSB-surrounding nuclear environment. Defects in a given repair pathway or inappropriate repair pathway choice can be exploited for synthetic lethal cancer therapy approaches, such as poly(ADP-ribose) polymerase 1 (PARP1) inhibition, which selectively kills HR-deficient cancers (Lord and Ashworth, 2017).

Recent advances in biochemical, biophysical, imaging and deep sequencing technologies have led to unprecedented insight into nuclear organization and its changes with time and/or in response to cell-intrinsic or -extrinsic perturbations (Rowley and Corces, 2018; Kempfer and Pombo, 2020). Not surprisingly, there is an intimate link between genome organization, DNA accessibility and the functional regulation of DNA transactions, such as transcription, replication and DNA repair (Dekker and Mirny, 2016; Misteli, 2020). A first and by now well-characterized barrier to DNA access is the chromatin fiber, in which DNA is wrapped around positively charged nucleosomes consisting of a histone octamer, which typically comprises two copies of the core histones H2A, H2B, H3 and H4. Depending on the cellular context, core histones can be replaced with specialized histone variants. The impact of nucleosome composition and remodeling as well as static and dynamic histone modifications on DSB repair has been extensively investigated and we refer the reader to several excellent reviews summarizing this work (Price and D’Andrea, 2013; Lebeaupin et al., 2015; Ferrand et al., 2021; Hauer and Gasser, 2017). How the three-dimensional (3D) organization of the chromatin fiber in nuclear space can affect DSB repair, and conversely, be affected by the latter, is significantly less well understood.

Using a combination of high-throughput sequencing-based conformation capture approaches and fluorescent *in situ* hybridization (FISH)-based imaging, higher order chromatin organization can be interrogated at the kilobase (kb) or nm-scale, revealing complex chromatin looping that is often tied to cell cycle phases or DNA transactions such as transcription or replication (Bickmore, 2013; Rao et al., 2014; Rowley and Corces, 2018; Misteli, 2020). Chromatin loops are both architectural and functional in nature, providing a platform for 3D genome compaction as well as regulatory interactions. The size of chromatin loops can range from tens of kb to several 100 kb, often containing loops within loops. Loops that are characterized by unique chromatin features are referred to as topologically associated domains (TADs). Recent advances have provided significant insight into the processes that promote and maintain TAD formation, which involves active loop extrusion supported by architectural proteins such as CTCF and the structural maintenance of chromosomes (SMC) cohesin complex (Fudenberg et al., 2017; Rao et al., 2017; Davidson and Peters, 2021). Single cell analyses demonstrate that TAD formation is highly dynamic and often only detectable in a small subset of cells at any given time (Finn et al., 2019). For detailed reviews of recent advances in our understanding of TAD formation and function we refer the reader to (Hansen et al., 2018; Szabo et al., 2019). Of note, individual TADs can segregate into larger chromatin domains, which differ in loop density as well as nucleosome composition and mobility (Janssen et al., 2018; Szabo et al., 2019). While the formation of such chromatin domains is associated with TAD-specific histone modifications, recent findings demonstrate that liquid phase separation may help organize the heterochromatin compartment and perhaps TAD organization more generally (Larson and Narlikar, 2018; Gibson et al., 2019; Sanulli et al., 2019).

In this review, we will describe recent insight into the orchestration of DSB repair in the context of nuclear topology. Specifically, we will discuss how DSB repair processes have adjusted to and in many cases adopted the organizing principles of the nucleus to ensure accurate lesion repair. Finally, we will briefly address the consequences of failed 3D genome maintenance for genome instability and disease.

## Location, Location Location – Chromatin Context Affects Repair Outcome

Large-scale interdisciplinary efforts such as the Encyclopedia of DNA elements (ENCODE) or the 4D Nucleome Projects have helped to map the composition of mammalian chromatin with remarkable resolution (Consortium, 2004; Dekker et al., 2017; Consortium et al., 2020). As we continue to obtain more refined insight into how DNA is organized into functionally and phenotypically distinct chromatin domains, it is time to revisit how these domains affect genome integrity.

### Impact of Chromatin Context on DNA Double-Strand Break Repair Pathway Choice

Our understanding of the many chromatin modifications that interface with DSB repair processes is growing continuously and has been extensively reviewed elsewhere (Dabin et al., 2016; Hauer and Gasser, 2017; Ferrand et al., 2021). Nevertheless, a systematic assessment of DSB repair outcome across the various distinct chromatin states that coexist in a single cell has been missing to date. The advent of CRISPR/Cas9 as an effective means to target DSB induction to any given genomic location provides an opportunity to address this knowledge gap. Recent work by van Steensel and colleagues pioneered this effort by combining a multiplexed genome editing approach with a reporter that can distinguish between the two major error-prone DSB repair pathways, MMEJ and NHEJ (Schep et al., 2021). By overlaying a highly quantitative, sequencing-based “DNA repair scar”-counting readout with existing epigenome data, comprehensive MMEJ and NHEJ repair maps were generated for chromatin contexts across >1,000 genomic locations. This work complements and extends previous genome-wide assessments of HR versus NHEJ usage using the AsiSI endonuclease, which cuts the human genome efficiently at <100 sites and revealed a preference for HR in transcribed genomic regions (Aymard et al., 2014). Notably, MMEJ, which like HR relies on the resection of broken DNA ends to expose patches of homology for break alignment and repair, was found to be more frequent in specialized heterochromatic chromatin environments marked by H3 trimethylated at K27 (H3K27me3) (Schep et al., 2021). Together, these findings suggest that despite a common initial end processing step, HR and MMEJ are differentially controlled by chromatin context, perhaps by regulating the shift from short-range resection to long-range resection generally associated with HR (Symington and Gautier, 2011; Scully et al., 2019).

Supporting a functional role for H3K27me3 in modulating DSB repair outcome, inactivation of EZH2, the histone methyltransferase responsible for most of its deposition, caused a shift in DNA repair away from MMEJ towards NHEJ (Schep et al., 2021). Moreover, EZH2 inhibition was recently shown to shift repair from HR to NHEJ in some ovarian cancer cell lines (Karakashev et al., 2020). Rather than being a lesion-specific effect of H3K27me3, the reduction in HR efficiency upon EZH2 inhibition was due to transcriptional de-repression of MAD2L2, a component of the Shieldin complex that counteracts DNA end resection (Karakashev et al., 2020). Consistent with the defect in HR, EZH2 inhibition selectively sensitized ovarian cancer cell lines with sufficiently high MAD2L2 levels to PARP inhibitors in both orthotopic and patient-derived xenografts. Seemingly in contrast to these findings, EZH2 inhibition has recently been associated with replication fork stabilization and PARPi resistance in BRCA2-deficient and hence HR-defective breast cancer cells (Rondinelli et al., 2017). EZH2-mediated destabilization of replication forks involved H3K27me3 mediated recruitment of the MUS81 endonuclease, thus coupling histone modification to replication fork protection. Together, these observations emphasize that a widely distributed mark of facultative heterochromatin such as H3K27me3 can have a complex impact on genome maintenance, which likely depends on genomic context as well as the type of DNA lesion. Consequently, manipulation of EZH2 resulted in cell line-specific, yet predictable outcomes in response to genotoxic therapy.

The macro-histone variant macroH2A1, which frequently colocalizes with H3K27me3 domains across the genome (Chen et al., 2014), has recently emerged as another modulator of DNA repair pathway choice (Ruiz et al., 2019; Sebastian et al., 2020). Specifically, macroH2A1 controls DSB repair via balanced expression of its two alternative splice variants, macroH2A1.1 and macroH2A1.2. MacroH2A1.1, which unlike macroH2A1.2 can bind poly (ADP-ribose) (PAR), interacts with the MMEJ effectors PARP1 and Ligase 3 in a PAR-dependent manner to facilitate MMEJ, whereas macroH2A1.2 promotes HR by facilitating BRCA1 recruitment to sites of DNA damage (Khurana et al., 2014; Kim et al., 2018). Deletion of macroH2A1.2 shifts repair towards MMEJ resulting in genome instability that is particularly pronounced at the macroH2A1-and H3K27me3-rich inactive X chromosome in female mouse fibroblasts (Sebastian et al., 2020). If and how macroH2A1 and EZH2 functions are related during DSB repair and/or replication stress remains to be determined. However, deregulation of H3K27me3 levels or macroH2A1 variant expression, and the associated HR defects, have both been linked to PARP inhibitor resistance, chromosomal abnormalities, and […] PARP inhibitor resistance and chromosomal instability in cancer cells in cancer cells (Khurana et al., 2014; Karakashev et al., 2020).

Together, these recent advances exemplify the impact of improved integrative analyses of chromatin composition on our understanding of genome maintenance. They further emphasize the need to i) consider functionally distinct proteoforms, often as the result of alternative splicing, and ii) distinguish lesion-specific from global effects of chromatin perturbation such as the epigenetic deregulation of repair factors.

### Chromatin Domains Guide DNA Replication

Recent work suggests that, much like distinct chromatin domains differentially affect DSB repair factor recruitment, they can modulate the initiation and progression of DNA replication, as well as the repair of stalled replication forks (Alabert et al., 2017; Aladjem and Redon, 2017; Bellush and Whitehouse, 2017). While the impact of chromatin on replication timing and DNA polymerase processivity is well described (Marchal et al., 2019; Klein et al., 2021), it was perhaps unexpected that chromatin composition can determine the choice of DNA replisome subunits. Comparative analysis of two replisome-associated proteins involved in the cellular response to replication stress, the translocase FANCM and the poorly characterized DONSON protein (Reynolds et al., 2017), uncovered the existence of distinct replisome complexes. While both proteins facilitate the repair of DNA interstrand crosslinks (ICLs) via a lesion traverse mechanism, FANCM-associated replisomes are most prevalent in late stages of S phase, generally induced by replication stress, and colocalize with a chromatin environment characteristic of late replicating and fragile DNA (Zhang et al., 2020). DONSON, on the other hand, appears to form a distinct replisome complex that is primarily responsible for ICL traverse in early S phase. Notably, FANCM and DONSON show the same bias in replication timing- and chromatin domain-association in cells without ICLs. How distinct chromatin environments regulate replisome composition remains to be determined, as does the functional relevance and potential clinical implications of having different replisomes act throughout S phase. Of note, defects in DONSON or FANCM manifest in microcephalic dwarfism and breast cancer susceptibility, respectively (Reynolds et al., 2017; Catucci et al., 2018). It will be interesting to investigate whether or not these distinct pathological outcomes relate to the observed differences in replication stress responses.

Altogether, we anticipate that continued, refined and comprehensive mapping of functionally distinct chromatin components, DNA repair outcome and genetic dependencies will provide a wealth of clinically actionable insight into repair mechanisms.

## DNA Double-Strand Break Repair Domains – New Insights Into Formation and Function

Beyond its role in modulating and regulating DNA transactions, chromatin shapes and defines the formation of functionally distinct, specialized nuclear environments. Recently, it has become apparent that DNA repair takes advantage of these features to form contained and often pathway-specific micro-environments, sequestering DNA lesions for reasons that remain to be fully investigated, but may help prevent illegitimate and potentially harmful repair events. Several novel concepts highlight and extend the impact of nuclear organization to genome maintenance and are discussed below.

### Chromatin Loop Extrusion: The DNA Repair Focus Revisited

Microscopically visible DNA damage response (DDR) foci are a striking feature of DSB repair (Rogakou et al., 1999). These foci generally reflect a single DNA lesion and its association with a plethora of often repair pathway-specific damage sensors and repair factors that can cover several hundred kilobases (kb) of lesion-surrounding DNA. At the heart of most DSB-associated chromatin changes is the phosphorylation of S139 on the histone H2A variant H2AX (referred to as γH2AX), orchestrated by early DNA damage signaling events involving one or more of three PI3K family kinases - ATM, ATR and DNA-PKcs (Rogakou et al., 1998; Smeenk and van Attikum, 2013). γH2AX facilitates the recruitment of key downstream repair effector proteins via the γH2AX-binding MDC1 scaffold protein (Smeenk and van Attikum, 2013). Although DSB-surrounding γH2AX chromatin domains have been mapped across the genome in response to numerous DNA damaging agents or endonucleases, the molecular basis that underlies the formation of up to megabase (Mb) size regions of γH2AX has long puzzled the field (Iacovoni et al., 2010). Of note, γH2AX domain boundaries were found to coincide with topologically associated domain (TAD) boundaries (Caron et al., 2012), and super-resolution light microscopy revealed that CTCF, a TAD boundary marker, is juxtaposed to γH2AX foci (Natale et al., 2017). Similar observations were reported for the 53BP1 repair factor, the recruitment of which depends on the RNF8/RNF168 E3 ubiquitin ligases, which in turn bind MDC1 (Hustedt and Durocher, 2016; Ochs et al., 2019). Together, these observations suggest that DNA repair domain formation is governed by high-order chromatin organization.

The organization of the genome into TADs involves ATP-dependent, active extrusion of DNA loops through a cohesin ring (Rao et al., 2017; Ganji et al., 2018; Davidson et al., 2019; Davidson and Peters, 2021). Cohesin consists of the SMC1-SMC3 heterodimeric adenosine triphosphatase (ATPase), the SMC protein partner RAD21 and either of the helical repeat proteins STAG1 or STAG2, and was originally identified as a mediator of sister chromatid cohesion. TAD loops are anchored by inverted CTCF sites, which terminate cohesion-mediated loop extrusion when encountered on opposing strands through yet to be determined mechanisms. For a detailed overview of cohesin function in TAD formation, we refer the reader to a number of excellent reviews (Fudenberg et al., 2017; Rowley and Corces, 2018; Yatskevich et al., 2019).

**FIGURE 1:**
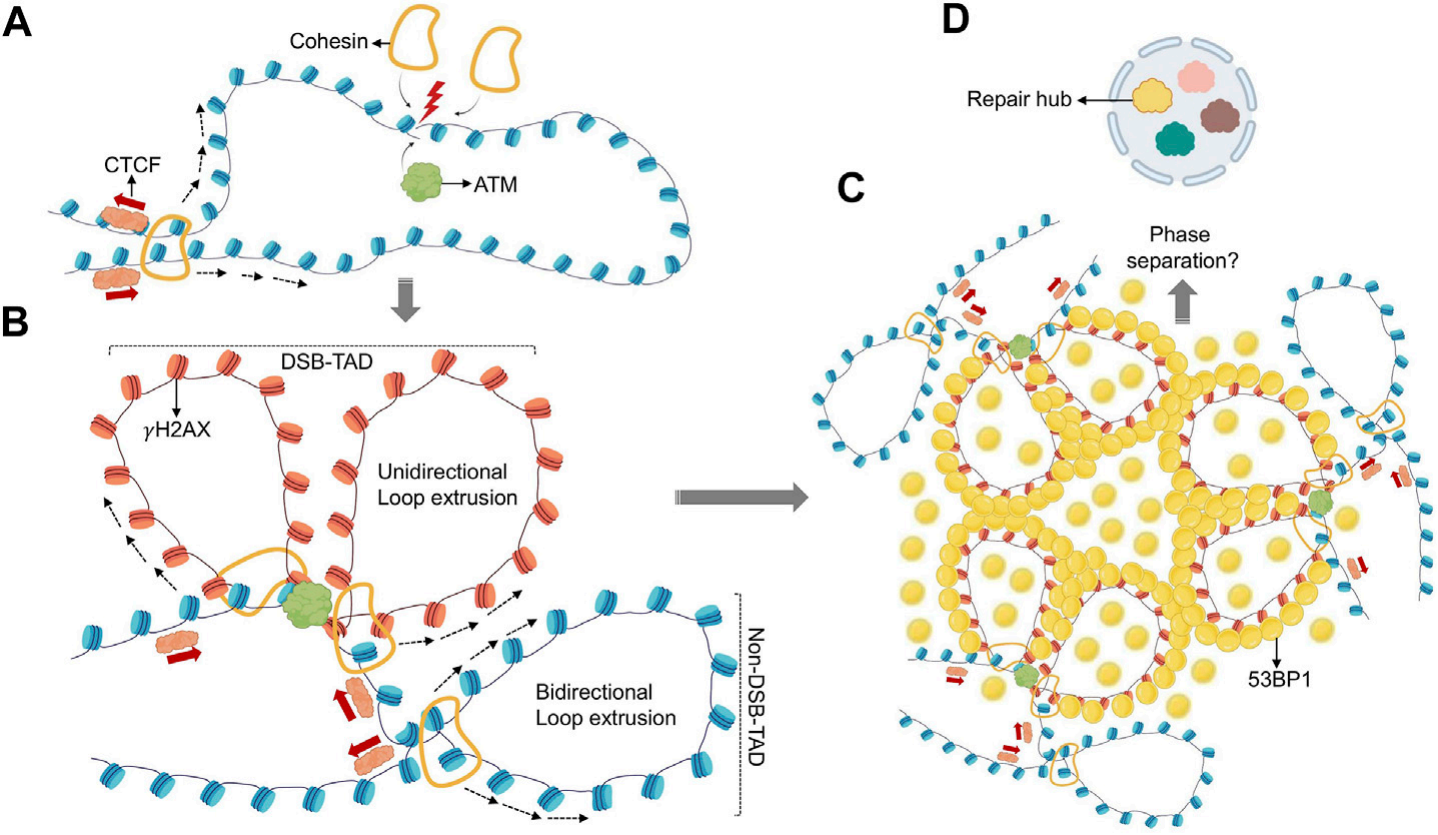
Role of loop extrusion in establishing repair domains. **(A)** DSBs initiate recruitment of ATM kinase and the cohesin complex. **(B)** DSB-associated cohesin anchors initiate unidirectional loop extrusion at both DSB ends, towards TAD anchors. ATM phosphorylates H2AX while nucleosomes are extruded (γH2AX nucleosomes are shown in red). Loop extrusion stops when existing TAD boundaires are encountered. This process generates a TAD-overlapping γH2AX domain. **(C)** γH2AX domains recruit 53BP1 repair factors creating similar, TAD-overlapping 53BP1 profiles. **(D)** 53BP1 mediated phase separation at TAD-associated 53BP1 domains may promote higher-order assembly of multiple 53BP1-TADs, and possibly multiple DSBs, to create spatially segregated repair hubs (yellow). Distinct nuclear subcompartments are symbolized in different colors.

Loop extrusion does not only facilitate the organization of TADs and chromatin domains, it can also facilitate the ligation of otherwise distal DNA ends. This was first described for the processes of VDJ and class switch recombination, which mediate the rearrangement and assembly of immunoglobulin (Ig) gene elements that are up to several 100 kb apart (Zhang et al., 2019a; Zhang et al., 2019b). Like TAD formation, VDJ recombination and class switching depend on CTCF-associated cohesin rings to allow for accurate ligation of matching gene elements (Ba et al., 2020; Dai et al., 2021). Notably, cohesin was found to accumulate at DSB sites other than the Ig locus, and its depletion resulted in genome instability (Ström et al., 2004; Ünal et al., 2004; Potts et al., 2006; Covo et al., 2010; Meisenberg et al., 2019). Recruitment of cohesin to DSBs was observed throughout the cell cycle, suggesting a function independent of sister chromatin adhesion or HR (Ström et al., 2004; Potts et al., 2006; Caron et al., 2012; Meisenberg et al., 2019). Together with the finding that γH2AX domains overlap with TADs (Caron et al., 2012; Natale et al., 2017), these observations point to a role for loop extrusion in the formation of DSB repair domains. Experimental support for the latter came recently from an elegant set of analyses combining chromosome conformation capture mapping, chromatin immunoprecipitation (ChIP) and AsiSI-mediated DSB induction (Arnould et al., 2021). A marked discrepancy was observed between the distribution of γH2AX and ATM, the kinase primarily responsible for DSB-induced H2AX phosphorylation (Arnould et al., 2021). In contrast to the TAD-sized γH2AX domains, ATM accumulation was restricted to the immediate vicinity of the DSB, suggesting that H2AX phosphorylation is not mediated by the linear spreading of the kinase across TADs. Using tightly controlled DSB synchronization and release combined with ATM inhibition and/or deletion of several cohesion subunits, the authors provide compelling evidence for a model in which H2AX-containing nucleosomes are rapidly phosphorylated as they actively pass by DSB-anchored cohesin. TADs thus delineate the boundaries of γH2AX chromatin domains in a manner that involves one-sided loop extrusion on either side of the break. Importantly, this process was conserved in yeast (Arnould et al., 2021), and the observed kinetics are consistent with previously reported rates of γH2AX foci assembly (Ochs et al., 2019). Moreover, DNA damage was shown to result in the ATM-dependent strengthening of existing TAD structures, perhaps as a mechanism to protect 3D genome integrity during DNA repair (Sanders et al., 2020). Together, these findings highlight how chromosome conformation and TAD-associated loop extrusion have been adopted by the DDR to ensure repair domain formation and genome maintenance (Figure 1).

### Phase Separation of Double-Strand Break Repair Domains?

In addition to structured chromatin organization, nuclear subdomains can be organized by physicochemical forces (Boeynaems et al., 2018). Examples of such domains include PML bodies, Cajal Bodies, nuclear speckles, and the nucleolus, which were proposed to behave as semifluid spheres suspended in semifluid nucleoplasm almost 2 decades ago (Handwerger et al., 2005). Experimental evidence for the physical nature of such assemblies was provided in 2009, when P granules (RNA and protein-containing bodies) were shown to display liquid-like properties and form by phase separation in *C. elegans* (Brangwynne et al., 2009). By definition, phase separation in biological systems occurs when a homogenous mixture of macromolecules such as proteins or nucleic acids in a solution spontaneously separate into a phases of distinct densities. In the context of chromatin, condensates can form either via bridging of nucleosome-binding proteins (polymer-polymer phase separation) or via multivalent interactions among soluble, chromatin-associated proteins (liquid-liquid phase separation) (Erdel and Rippe, 2018; Miné-Hattab and Taddei, 2019). In the case of liquid-liquid phase separation, which is the focus of this section, the dense phase has liquid-like properties, no fixed stochiometry and accumulates certain macromolecules. Since the non-dense phase is depleted of said macromolecules, it allows the dense phase to attain a compartment-like status. Phase-separated liquid condensates can eventually form more solid-like states exhibiting different material properties, such as dynamic liquid-like droplets or less dynamic gels and solid amyloids (Banani et al., 2017; Miné-Hattab and Taddei, 2019).

**FIGURE 2:**
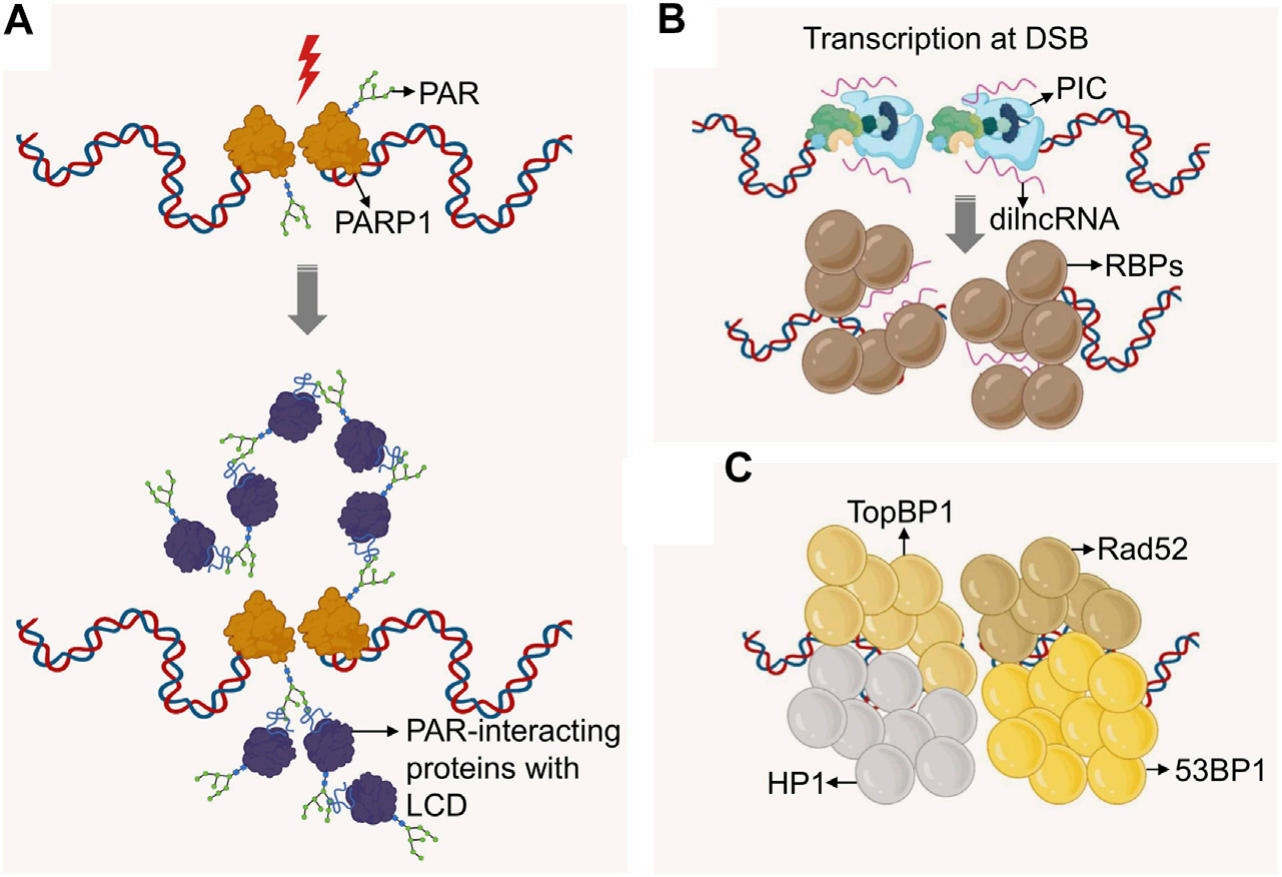
Formation of DNA repair domains via phase separation. **(A)** DSBs recruit PARP1, which mediates DSB-proximal PARylation, attracting PAR-binding proteins, many of which contain Low Complexity Domains (LCD). The latter promote molecular crowding and concomitant phase separation. **(B)** DSBs recruit RNA Polymerase II, which initiates transcription at the DSB site to generate dilncRNAs. These RNAs are bound by IDR-containing RNA binding proteins (RBPs) which can drive phase separation. **(C)** Protein modifications at DSBs recruit proteins such as 53BP1, TopBP1, Rad52 and HP1, all of which were shown to form higher order condensates via phase separation. If these domains are distinct or can be fused remains to be determined. 53BP1 may further promote phase separation via its binding to dilncRNA.

Studies looking at the protein composition of phase-separated biological condensates suggest multivalency of adhesive domains and linear motifs as defining features of proteins that drive phase separation. Prominent examples are intrinsically disordered regions (IDRs) and Low Complexity Domains (LCDs). Of note, recent work from the Narlikar and Karpen labs demonstrated that phase separation is also an organizing principle for chromatin domains, particularly heterochromatin (Larson et al., 2017; Strom et al., 2017; Larson and Narlikar, 2018). It may thus be not surprising that phase separation was found to contribute to the formation of DNA repair micro-environments. Underlying mechanistic insight and possible consequences for genome maintenance are discussed below, separated by the phase-separating properties of primary responders or effectors of the DNA damage response (Figure 2).

#### Phase Separation by Poly (ADP-Ribose)

PARP1 is an abundant nuclear protein that attaches a negatively charged (PAR) polymer to itself and to multiple target proteins. This modification is one of the earliest events in the DNA damage response against a wide variety of DNA lesions (Kraus, 2020). Consistent with this, PARylation has been implicated in the repair of single-strand breaks (SSBs), DSBs, the stabilization of DNA replication forks and the modification of the DNA damage-associated chromatin (Ray Chaudhuri and Nussenzweig, 2017). While PARP1 itself has no IDRs, its activation at sites of DNA damage was found to promote transient phase separation via the formation of PAR chains. PAR chains act as a molecular scaffold for the assembly of proteins with disordered or low complexity domains, thereby initiating demixing of distinct liquid phases to achieve dynamic intracellular compartmentalization. Two types of LCDs participate in this process: positively charged arginine–glycine–glycine (RGG) repeats, which act as a PAR sensor, and prion-like protein domains, which amplify PAR-seeded liquid demixing (Altmeyer et al., 2015). This process appears to reflect a general mechanism to dynamically reorganize the soluble nuclear space in response to DNA lesions (Figure 2A). Recent work has implicated the highly disordered RGG containing Fused in Sarcoma (FUS/TLS) protein in PAR-seeded liquid demixing (Singatulina et al., 2019). FUS, together with EWS and TAF15, is a member of the FET family and one of the most abundant and highly PARylated nuclear RNA-binding proteins (Britton et al., 2014; Singh et al., 2015; Zhen et al., 2017). FUS condensates have liquid-like properties, the dynamics and structure of which are affected by pathogenic mutations as well as LCD phosphorylation (Patel et al., 2015; Monahan et al., 2017; Murray et al., 2017). Upon DNA damage, the C-terminal RGG repeats of FUS form repair domains in response to PARP activity in a transient and reversible manner (Singatulina et al., 2019). PAR-seeded liquid demixing may thus facilitate the compartmentalization of damaged DNA, and its functional relevance for the DDR is a subject of intense investigation.

#### Phase Separation by RNA

Analogous to PAR chains, nucleic acids were shown to seed phase-separated structures by recruiting IDR-containing RNA binding proteins (RBPs). Non-coding RNAs (ncRNAs) form molecular scaffolds that connect multiple RBPs into a dynamic network of phase separated droplets (Lin et al., 2015; Aumiller et al., 2016; Pessina et al., 2019; Guo et al., 2021). Messenger RNA (mRNA) was also found to form phase-separated droplets. However, in this case the seed involved specific 3D structures through complementary RNA base pairing (Langdon et al., 2018). Of note, growing evidence points to DNA damage-induced transcription of non-coding RNA at DNA break sites (Sebastian and Oberdoerffer, 2017; Zong et al., 2020). These DNA damage induced long non-coding RNAs (dilncRNA) were found to be necessary for DNA damage response (DDR) focus formation (Francia et al., 2012), form transient RNA:DNA hybrids (Wahba et al., 2013; Ohle et al., 2016), and regulate the extent of end resection and consequently HR. Recently, it was shown that the induction of DSBs resulted in the assembly of functional promoters that include a complete RNA polymerase II preinitiation complex, MED1 and CDK9 (Pessina et al., 2019). Mediator and RNA polymerase II clusters are known to associate in transcription-dependent condensates (Cho et al., 2018), and depletion or inactivation of these factors caused a reduction in DDR foci. Moreover, dilncRNAs drove molecular crowding of DDR proteins, such as 53BP1, into foci that behave like phase-separated condensates (Pessina et al., 2019). Given that phase separation has been proposed as a mechanism for transcription control (Hnisz et al., 2017), a similar role in the DDR may provide an intriguing rationale for DSB-associated transcripts (Figure 2B).

#### Phase Separation by DNA Repair Factors

Phase separation can also be mediated by DNA repair proteins. The NHEJ effector and chromatin binding protein 53BP1 was recently shown to condensate into repair domains that are dynamic and show droplet‐like behavior (Figure 2C). Repair domain formation by 53BP1 undergoes frequent fusion and fission events, is highly sensitive to changes in osmotic pressure, temperature, salt concentration and the disruption of hydrophobic interactions, consistent with liquid demixing (Kilic et al., 2019). Light-induced optoDroplet formation experiments (Taslimi et al., 2014; Shin et al., 2017) combined with 53BP1 mutagenesis suggest that a C-terminal multivalent domain as well as the C-terminal BRCT domain are sufficient for 53BP1 phase separation properties. The implication of the BRCT domain is intriguing as other BRCT-containing protein such as BRCA1 did not appear to phase-separate, suggesting sequence specificity and/or more complex organizing principles. Providing functional insight into possible roles of DNA damage-induced phase separation, the tumor suppressor protein p53 was found enriched within 53BP1 repair domains, and conditions that perturb 53BP1 phase separation negatively affected 53BP1‐dependent activation of p53 (Kilic et al., 2019). However, a direct role for 53BP1-mediated droplet formation in the repair of DSBs has not been identified to date.

53BP1 foci have regulatory functions beyond the immediate repair of DSBs. Cells that carry replication stress-associated DNA damage, such as lesions resulting from under-replicated DNA, into the next cell cycle form so called 53BP1 nuclear bodies (Harrigan et al., 2011; Lukas et al., 2011). Like DSB-induced 53BP1 foci, 53BP1 nuclear bodies were sensitive to osmotic stress, indicative of phase separation properties (Kilic et al., 2019). Nuclear body formation appears to inhibit repair in G1 to facilitate templated, RAD52-mediated repair of the lesion in the next S phase (Lezaja and Altmeyer, 2018; Spies et al., 2019). Notably, RAD52 was shown to form liquid droplets in *Saccharomyces cerevisiae* (Oshidari et al., 2020). RAD52 droplets cooperate with DNA damage-inducible intranuclear microtubule filaments to promote the clustering of DNA damage sites and facilitate HR (Oshidari et al., 2020). Recent studies suggest that RAD52 may be a “client” rather than a “scaffold” for liquid droplets, pointing to additional factors involved in their formation (Miné-Hattab et al., 2021). It will be interesting to determine if a dynamic transition exists between 53BP1 nuclear bodies and RAD52 droplet formation, which may help regulate repair activity at 53BP1 nuclear bodies in a cell cycle-dependent manner.

A number of other DSB repair-associated proteins have been reported to exhibit phase separation properties (Figure 2C). A notable example is heterochromatin protein 1 (HP1), which can associate with sites of DNA damage to aid the recruitment of 53BP1 and RAD51 (Alagoz et al., 2015). HP1 has been implicated in the evolutionarily conserved, liquid-liquid phase separation of heterochromatin domains (Larson et al., 2017; Strom et al., 2017; Sanulli et al., 2019; Wang et al., 2019). However, both the precise nature of HP1 subcompartment formation and its potential role at DSBs remain to be determined (Chiolo et al., 2011; McSwiggen et al., 2019; Erdel et al., 2020). More recently, the ATR activator TopBP1 was shown to self-assemble into micron-sized condensates. Single amino acid substitutions of key residues in the ATR-activation domain of TopBP1, which also contains IDRs, disrupt TopBP1 condensation and, consequently, ATR/Chk1 signaling and replication fork stalling (Frattini et al., 2021). Of note, DSB-dependent formation of early DDR events such as γH2AX and MDC1 foci did not exhibit liquid-like properties (Kilic et al., 2019), pointing to distinct and likely dynamic modes of DSB micro-environment organization. The latter is further consistent with the seemingly independent and/or complementary initiation of phase separation initiation at DNA lesions via either PAR, RNA or DNA repair factors.

### Coordination Between Topologically Associated Domains and Phase Separation in DNA Repair

Recent evidence suggests that the formation of phase-separated repair environments is tightly linked to TAD-associated repair micro-domains. Using super-resolution microscopy, Lukas and colleagues were able to provide unprecedented insight into repair domain formation by 53BP1 (Ochs et al., 2019). Specifically, 53BP1 and its interacting factor RIF1 were found to form an autonomous functional module that stabilizes 3D chromatin topology at sites of DNA breakage. This process involves the sequential accumulation of 53BP1 at TAD-associated, compact chromatin, followed by RIF1 accumulation at the boundaries between these domains. The alternating distribution of 53BP1 and RIF1 was found to stabilize neighboring TADs into a higher-order arrangement surrounding a single DSB. Depletion of 53BP1 or RIF1 led to the de-condensation of DSB-surrounding chromatin and aberrant spreading of DNA repair factors. Of possible functional relevance, depletion of either protein also resulted in hyper-resection of DNA ends (Ochs et al., 2019).

Interestingly, topological distortions of 53BP1 domains could also be observed upon depletion of cohesin (Ochs et al., 2019). Together with recent insight into TAD-dependent DDR focus formation, these findings suggest a staged model wherein TAD structure dictates γH2AX foci formation, which in turn promotes their DNA repair-independent, 53BP1-mediated higher-order assembly, perhaps in a process that involves phase separation (Figure 1). More work is needed to understand the implications of this 3D re-organization of the DSB-proximal chromatin micro-environment for DNA repair, but the observed changes in DNA resection point to a role in the regulation of DSB repair pathway choice.

## DNA Lesions on the Move - Aggregation of Double-Strand Break in Three Dimensions

**FIGURE 3:**
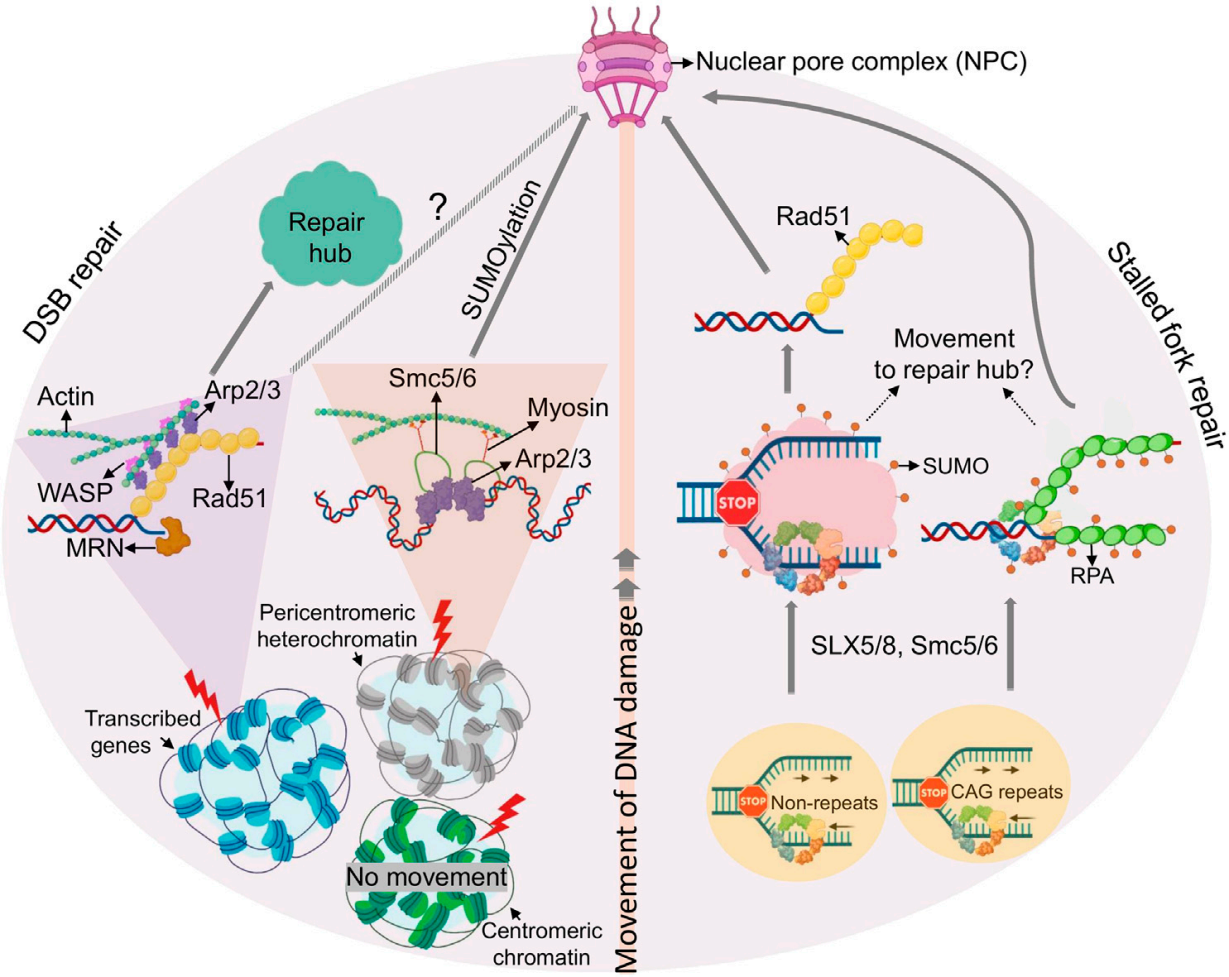
Movement of DSBs and damaged replication forks. Emerging principles of DSB (left) or replication fork mobility (right) are shown. Clockwise from the bottom: Mechanisms of movement are different for DSBs induced in different chromatin environments (see text for details). In brief, DSBs in transcriptionally active chromatin load Rad51 after end resection and move to repair hubs facilitated by actin polymerization. DSBs in pericentromeric heterochromatin initiate resection, but relocate away from hetercochromatin via an SMC5/6 dependent process to facilitate RAD51 loading. Pericentromeric DSBs, and perhaps DSBs within transcribed DSBs regions, are then targeted to the NPC in a manner that involves SUMOylation, myosin, and actin. Counterclockwise from the botton: Stalled replication forks relocate to the NPC in a process involving SUMOylation by Smc5/6 and/or SLX5/8 (shown as red dots). Forks in repetitive sequences are targeted to the NPC via SUMO-RPA, prior to Rad51 loading, whereas arrested forks at non-repetitive genomic loci are targeted to NPC after RAD51 loading.

Both TAD- and phase separation-associated repair domain formation can be observed at a single DNA lesion. However, the process of 53BP1-dependent clustering of multiple TAD domains, as well as the inherent biophysical properties of liquid demixing raise the possibility of a higher-order organization of multiple DNA lesions into a single repair “super-focus.” Moreover, recent advances in high-resolution live cell microscopy and targeted genome manipulation have uncovered compelling evidence for directed movement of DNA lesions to form aggregates. Although the phenomenon of DSB clustering has been observed in yeast and mammalian cells almost 2 decades ago (Lisby et al., 2003; Aten et al., 2004), until very recently little was known about the underlying mechanistic forces and possible functional relevance. In the following we will discuss novel insight into DSB mobility and one of the pre-eminent nuclear environments they congregate at, the nuclear pore (Figure 3).

### Movement and Clustering of DNA Lesions

Multiple lines of evidence suggest that DSB mobility can be an active process. Almost 10 years ago, homology search of a single DSB in yeast was shown to involve DNA end resection and RAD51-depedent DSB movement (Dion et al., 2012; Miné-Hattab and Rothstein, 2012). RAD51-coated DNA can explore a larger nuclear volume than undamaged DNA, which is thought to facilitate homologous pairing and repair. Mean square displacement (MSD) analyses, which plot the average of the squared distances that a particle has travelled against increasing time intervals, suggested that increased DSB mobility was due to an increase in the radius of confinement, rather than a change in the diffusion coefficient of the damaged locus, pointing to a role for chromatin reorganization in this process (Miné-Hattab and Rothstein, 2012; Hauer and Gasser, 2017). DSB mobility has since then emerged as a complex phenomenon that depends on various factors, including cell cycle phase and DSB location (Kalousi and Soutoglou, 2016; Smith and Rothstein, 2017). Telomeric DSBs, for example, are more mobile than the undamaged chromatin (Dimitrova et al., 2008), whereas UV laser microirradiation or endonuclease-mediated induction of DSBs outside of telomeres show limited mobility (Kruhlak et al., 2006; Soutoglou et al., 2007; Roukos et al., 2013). Such discrepancies have sparked intensive efforts to better understand the molecular mechanisms that drive DSB mobility, both in yeast and higher organisms. Much of the initial progress came from studies of broken telomeres, which are relatively easy to monitor in living cells. Telomeric DSBs can result from telomere deprotection and are subject to repair by NHEJ (de Lange, 2018). In mammalian cells, the NHEJ effector 53BP1 was found to promote not only repair but also mobility of broken telomere ends, together with the linker of nucleoskeleton and cytoskeleton (LINC) complex and dynamic microtubules (Lottersberger et al., 2015). 53BP1/LINC-dependent DSB mobility was not limited to telomeres, but was also observed upon irradiation-induced DNA damage. Given the role for 53BP1 in DSB-associated phase separation described in *Phase Separation by DNA Repair Factors*, it will be interesting to determine if the latter contributes to or complements the mechanic movement forces provided by 53BP1, the LINC complex and microtubules. Precedent for a coordination between phase separation and active movement comes from the observation that RAD52 droplets can cooperate with microtubule filaments to promote DSB clustering and repair (Oshidari et al., 2020). While the functional relevance of 53BP1-mediated DSB clustering remains to be established, this process may help restore proximity of DNA ends that have lost their proper interaction and thereby counteract ectopic repair. However, with increasing DNA damage, aberrant end pairing can have fatal outcomes, as evidenced by aberrant telomere end fusions that result in dicentric chromosome formation (Lottersberger et al., 2015).

A distinct type of telomeric break movement has been described in the context of alternative lengthening of telomeres (ALT), an HR-dependent process to maintain telomeres in the absence of telomerase, which is active in ∼15% of cancer types. ALT-associated homologous chromosome synapsis was found to depend on long-range DSB mobility and aggregation into multi-telomere clusters (Cho et al., 2014). Much like HR-prone DSBs in yeast, this process required RAD51, although it further involved the protein dimer Hop1/Mnd1, which also mediates homologous chromosome synapsis during meiosis. Moreover, ALT telomeric DSBs show evidence for directed motion based on MSD analysis (Cho et al., 2014), while DSB movement in yeast was found to be consistent with confined Brownian motion (Dion et al., 2012; Miné-Hattab and Rothstein, 2012). Together, these findings underline the context dependence of telomeric DSB movement.

Telomeres present a unique chromatin environment and the implications of break mobility outside of telomeric regions have only recently been uncovered in vertebrates. In a mass spectrometry approach in *Xenopus* extracts, Gautier and colleagues identified nuclear actin, the actin-nucleating complex ARP2/3, β-actin and the ARP2/3 activator WASP as novel, chromatin-associated DSB repair effectors (Schrank et al., 2018). While DNA damage-induced actin polymerization was reported previously (Belin et al., 2015), little was known about its role in DSB repair and/or at broken DNA. Using the mammalian AsiSI endonuclease system described earlier (Aymard et al., 2014), ARP2/3 and WASP were found to preferentially accumulate at HR-prone DSB sites. Consistent with the latter, HR but not NHEJ efficiency was impaired upon inhibition of actin nucleation, or the depletion of WASP or the nucleation factors FORMIN-2 and SPIRE-1/SPIRE-2. Moreover, nuclear actin polymerization was found to be required for G2-restricted migration of a subset of DSBs and their aggregation into sub-nuclear clusters. Like mobilty in yeast and at ALT telomeres, AsiSI-induced DSB movement was initiated by DNA end resection (Dion et al., 2012; Miné-Hattab and Rothstein, 2012; Cho et al., 2014; Schrank et al., 2018). Interestingly, ARP2/3 loading was found to enhance end resection and RAD51 loading at AsiSI-induced DSBs in a positive feedback loop (Schrank et al., 2018). DSB movement at HR-prone AsiSI DSBs was further found to be consistent with confined Brownian motion, similar to yeast (Schrank et al., 2018). A role for actin nucleation in the movement of yeast or ALT DSBs remains to be demonstrated.

It should be noted that there is some debate as to when during the cell cycle DSBs cluster. Seemingly in contrast to the findings by the Gautier lab, enhanced DSB clustering was first identified in G1 cells (Aten et al., 2004). Preferential aggregation in G1 was confirmed more recently using Hi-C chromosome conformation capture of AsiSI-dependent DSBs (Aymard et al., 2017), although clustering was similarly restricted to HR-prone break sites. DSB clustering in G1 coincided with delayed DSB repair and was dependent on the MRN complex, FORMIN-2 and the LINC complex, consistent with resection-mediated active movement. Given the identical DSB source (AsiSI), discrepancies in the timing of DSB clustering may reflect distinct experimental readouts, such as the resolution of Hi-C versus live cell imaging assays, which is likely to detect significantly smaller aggregates in the case of Hi-C. It will be interesting to determine if distinct “micro” and “macro” aggregate sub-types exist, and how they may differentially contribute to the DDR. One intriguing hypothesis is that transitions in aggregate sub-type may help control repair kinetics during the cell cycle, ensuring HR in S/G2, but preventing HR in G1.

Much like HR, actin-mediated DSB mobility is not restricted to transcribed genes. HR in highly repetitive DNA, such as pericentromeric heterochromatin relies on specialized mechanisms to prevent aberrant recombination events. In *Drosophila melanogaster*, this is achieved by relocalization of DSBs to the nuclear periphery (Chiolo et al., 2011). While proteins responsible for the initial steps of end resection are rapidly recruited within heterochromatin, RAD51 remains excluded, thus preventing homology search and completion of HR. RAD51 loading instead requires resected heterochromatic DSBs to move to the nuclear periphery in a process that involves the SMC5/6 SUMO E3 ligases (Chiolo et al., 2011; Ryu et al., 2015). A similar process has been observed in yeast and at the repetitive rDNA, and has been extensively reviewed elsewhere (Jalal et al., 2017). More recently, Chiolo and colleagues demonstrated that, much like mammalian AsiSI-induced, HR-prone DSBs, the movement of heterochromatic breaks to the nuclear periphery in *Drosophila* requires actin filament formation and the Arp2/3 complex (Caridi et al., 2018). However, while ARP2/3 mediated actin nucleation appears to be sufficient for mobility and clustering of non-heterochromatic DSBs in mammalian cells (Schrank et al., 2018), DSBs within *Drosophila* heterochromatin further require nuclear myosin, which associates with Smc5/6 proteins to initiate movement (Caridi et al., 2018). Notably, two phases of motion have been described for heterochromatic lesions in Drosophila: confined Brownian motion within the heterochromatin domain, and directed motion towards the NPC, outside of heterochromatin (see Figure 3) (Caridi et al., 2018; Miné-Hattab and Chiolo, 2020). Given that Arp2/3 promotes non-directed motion of mammalian HR-prone DSBs, actin appears to be able to support both types of motion, implicating additional mobility modulators or species-specific differences. Together, these findings suggest that HR-prone DSBs can initiate movement irrespective of genomic context, but mobility may require additional accessory factors, depending on where the DSBs occur. Why HR-prone breaks move preferentially compared to non-HR prone lesions remains an open question, but further points to a critical role for end resection in this process.

Notably, not all DSBs within compacted chromatin initiate movement, even if they are destined for HR. While DSBs relocalize in the context of pericentromeric heterochromatin as described above, the same does not appear to be true for DSBs in centromeric chromatin, which carries distinct epigenetic marks and occupies distinct nuclear subdomains. The precise nature of this discrepancy remains to be investigated (Tsouroula et al., 2016). Consistent with the findings by the Gautier lab (Schrank et al., 2018), DSBs in mammalian pericentromeric heterochromatin were found to be positionally stable in G1, where they recruit NHEJ factors, while their resection in S/G2 promoted relocalization away from heterochromatin to allow for RAD51 binding and HR. Centromeric chromatin, on the other hand, was accessible to HR and NHEJ factors throughout the cell cycle and did not require DSB movement for their repair (Tsouroula et al., 2016).

Altogether, these findings add significant new insight into the complexity of the forces that drive DSB mobility, and often DSB clustering, across species, and further place this process at a central position in the control of repair outcome and genome maintenance.

### On the Move – But Where to? The Nuclear Pore as a Repair Hub

Once movement of a DNA lesion is initiated, a common theme across species is its relocalization to the nuclear periphery, and specifically the nuclear pore complex (NPC). Movement of DSBs to the NPC has been extensively reviewed elsewhere (Freudenreich and Su, 2016; Schrank and Gautier, 2019). In the following, we will focus on new insight describing the nuclear pore as a specialized repair microenvironment for aberrant replication forks.

Various studies in yeast have shown that collapsed replication forks localize to the NPC in a process that is reminiscent of the movement of DSBs described in *Drosophila* (see *Movement and Clustering of DNA Lesions*, (Chiolo et al., 2011; Ryu et al., 2015)) and similarly depends on SUMO E3 ligases (e.g., SLX5/SLX8, SMC5/6) (Nagai et al., 2008; Freudenreich and Su, 2016; Whalen et al., 2020). Relocation of poly-SUMO-modified arrested forks impedes fork repair by HR until anchorage at the NPC allows for SUMO removal by the SENP SUMO protease Ulp1 and the proteasome, which in turn promotes resumption of DNA synthesis by HR via a process known as Recombination-Dependent Replication (RDR) (Kramarz et al., 2020). Regions undergoing RDR-associated DNA synthesis are prone to chromosomal rearrangements (Lambert et al., 2010; Mizuno et al., 2013), providing a rationale for the spatial segregation of arrested forks within nuclear space.

Relocation of arrested replication forks was observed both at replication obstacles within a unique genomic context and at inherently difficult to replicate repetitive loci. However, the underlying mechanisms appear to be distinct. The relocation of forks collapsed at expanded CAG repeats requires nuclease activities to engage SUMO-RPA onto ssDNA, which prevents Rad51 loading (Whalen et al., 2020). Their anchorage to the NPC is required for RPA removal and efficient Rad51 loading, providing a means to constrain recombination at stalled or collapsed forks until it is required for fork restart. In contrast, relocation of a unique fork block to the NPC was found to occur *after* RAD51 loading, which may be tolerated due to the less recombinogenic nature of a non-repetitive DNA (Kramarz et al., 2020). While both relocation events require SLX5/8-mediated SUMOylation, SUMO-RPA accumulation appears to be specific for lesions in repetitive DNA (Whalen et al., 2020). If and how chromatin composition in these distinct genomic contexts determines whether or not RPA is SUMOylated remains to be determined. Altogether, SUMO-based NPC anchorage mechanisms spatially segregate HR events at broken forks at various steps in the repair process, but with the common goal to constrain recombination until it can be safely executed to allow fork restart.

Extending the parallels between the movement of DSBs and stalled or broken forks, recent work by the Cesare lab identified a role for nuclear actin in fork movement to the nuclear periphery (Lamm et al., 2020). Using live and super-resolution imaging, nuclear F-actin was shown to polymerize in response to replication stress in an ATR kinase-dependent manner that further involved WASP and ARP2/3. Much like at heterochromatic DSBs in *Drosophila*, F-actin and myosin promoted the mobility of stressed replication foci. Actin was further required to resolve replication stress and suppress chromosome and mitotic abnormalities. Finally, nuclear F-actin was detected in human tumor xenografts upon replication stress, indicating disease relevance (Lamm et al., 2020; Lamm et al., 2021). Beyond the response to replication stress, actin dynamics were recently shown to facilitate replication initiation in unperturbed cells by promoting the loading of cyclin-dependent kinase (CDK) and proliferating cell nuclear antigen (PCNA) onto chromatin (Parisis et al., 2017). If the latter contributes to replication re-initiation at stalled forks upon their F-actin-dependent relocalization remains to be investigated.

Not surprisingly, difficult-to-replicate ALT telomeric DNA also localized to the nuclear periphery in an actin-polymerization-dependent manner in response to replication stress (Lamm et al., 2020). Similarly, in yeast, subtelomeric DSBs were found to move to the nuclear pore for repair via break-induced replication, a means to repair single-ended DSBs often associated with the ALT pathways (Chung et al., 2015; Dilley et al., 2016; Oshidari et al., 2018). Movement of subtelomeric breaks require kinesin motor proteins and microtubule polymerization, extending the repertoire of motor proteins at stalled forks beyond F-actin (Chung et al., 2015; Oshidari et al., 2018). Of note, unlike stalled replication forks, endonuclease-mediated DSBs at subtelomeric regions in ALT cells were shown to aggregate predominantly in ALT-associated PML nuclear bodies, which may promote clustering and recombination of telomere ends (Cho et al., 2014) (see also *Movement and Clustering of DNA Lesions*). It will be interesting to determine what accounts for the differential targeting of these distinct, ALT telomere-associated DNA lesions.

Altogether, these recent advances highlight the importance of the nuclear pore as a repair-permissive microenvironment that supports the resolution of both DSBs and replication stress. Future work will need to uncover why the NPC presents a preferential “meeting point” for DNA lesions, and why this environment appears to be selectively associated with HR.

## 3D Genome Organization-Related Genome Instability in Cancer and Disease

Having reviewed the importance of 3D nuclear organization in the context of genome maintenance and accurate DNA repair, it must be noted that the spatial arrangement of chromatin has a direct effect on genome instability, often dictating the outcome of translocations and other aberrant repair events (Hakim et al., 2012; Roukos et al., 2013; Roukos and Misteli, 2014). In the following, we will discuss how nuclear topology can affect mutagenesis and the development of disease.

### Topologically Associated Domains, DNA Double-Strand Breaks and Genome Instability

First experimental evidence that TADs, and particularly the anchor regions of these chromosome loops, may pose a threat to genome integrity came from the Nussenzweig lab in 2017 (Canela et al., 2017). Loop anchor regions, as defined by both Hi-C contact maps and ChIP for the anchoring factors CTCF and cohesin (RAD21), were found to be a hot spot for Topoisomerase 2 (TOP2)-mediated DSB breakage. Consistent with this, TOP2B, one of two TOP2 isoforms in mammalian cells, has been shown to physically interact with CTCF and cohesin (Witcher and Emerson, 2009; Uusküla-Reimand et al., 2016) and is enriched in CTCF/cohesin-bound genomic regions (Madabhushi et al., 2015; Uusküla-Reimand et al., 2016). Of note, CTCF/TOP2-associated DSBs at TAD boundaries frequently involve breakpoint clusters that are commonly translocated in cancer, and were shown to drive cell-type- and tumor-specific chromosomal translocations (Canela et al., 2019; Gothe et al., 2019). Thus, loop anchors appear to be genomic fragile sites that can generate DSBs and chromosomal rearrangements. Moreover, these regions are particularly sensitive to treatment with the TOP2 poison and chemotherapeutic agent etoposide, which stabilizes the TOP2 cleavage complex and thus enhances DSB formation. As a result, etoposide treatment is frequently associated with recurrent chromosome translocations involving TAD boundaries in therapy-related myeloid leukemias (t-AML) (Wright and Vaughan, 2014).

**FIGURE 4:**
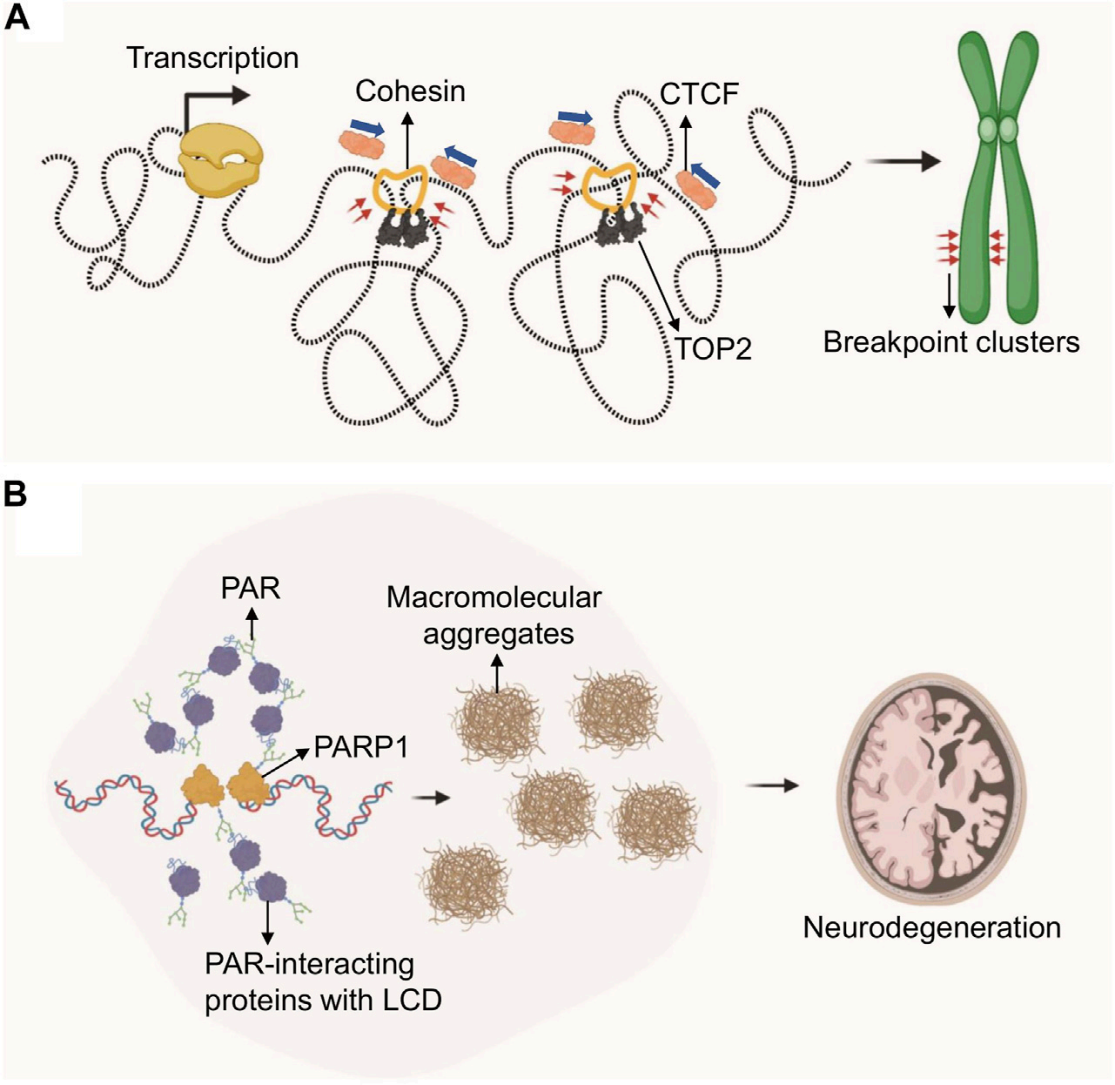
Role of genome architecture in genome instability and disease. **(A)** Loop extrusion by cohesin creates tortional stress on DNA, which is relieved by topoisomerases. TOP2 inhibition via etoposide covalently traps TOP2 at TAD boundaries which generates DSBs in the presence of transcription, ultimately resulting in chromosomal translocations. Etoposide–treatment in cancer is frequently associated with recurrent chromosomal translocations at TAD-boundary-associated breakpoint clusters. **(B)** DNA damage-driven, protein-rich biomolecular condensates are linked to neurodegeneration in A-T or ATLD patients. DDR defects in these patients cause PARP1 hyperactivation and PAR chain accumulation. PAR-dependent protein aggregates are found in A-T patient cerebellum.

While TOP2 chromatin localization and trapping at CTCF/cohesion anchors was shown to be independent of transcription (Canela et al., 2017), the conversion of trapped TOP2 cleavage complexes into DSBs correlates with transcriptional output and directionality (Canela et al., 2019; Gothe et al., 2019). Consistent with the latter, genes that recurrently translocate to drive leukemias are highly transcribed and are enriched at loop anchors (Gothe et al., 2019). Transcription and 3D chromosome folding thus pose a joint topological threat to genomic stability and are key contributors to the occurrence of genome rearrangements that drive cancer (Canela et al., 2019; Gothe et al., 2019) (Figure 4A).

In addition to topoisomerase poisons, mild replication stress was also able to trigger DNA fragility at TAD boundaries, often mapping to difficult to replicate genomic regions known as common fragile sites (CFSs) (Sarni et al., 2020). This effect was particularly pronounced at transcribed large genes that span TAD boundaries and coincided with a delay in replication timing (Sarni et al., 2020). Of note, replication domain boundaries overlap TAD boundaries, suggesting that TADs are regulatory units of replication timing (Pope et al., 2014; Marchal et al., 2019), and providing a rationale for the unique sensitivity of TAD boundaries to replication delays. It will be interesting to determine if this process further involves TOP2-mediated DSB induction.

### Phase Separation in Neurodegenerative Disease

While DNA lesion-associated phase separation is emerging as an integral and dynamic aspect of the DNA damage response, recent work by Paull and colleagues suggests that PAR-seeded liquid demixing may ultimately result in insoluble protein-rich biomolecular condensates observed in the cerebellar neurodegenerative disorder associated with ataxia-telangiectasia (A-T) (Lee et al., 2021) (Figure 4B). A-T is caused by the loss of ATM kinase, and hypomorphic mutations in the MRE11 repair factor can cause the related A-T-like disorder (ATLD), implicating a prominent role for DNA damage in disease progression (Taylor et al., 2004; Regal et al., 2013). While malignancy and immunodeficiency of A-T and ATLD patients is readily explained by DNA repair defects, the source of neurotoxicity in these patients remains poorly understood (Shiloh, 2020). Genetic ATM separation-of-function mutations previously demonstrated that ATM mutations associated with a loss of its activation by oxidative damage resulted in widespread protein aggregation (Guo et al., 2010; Lee et al., 2018). In seeking to understand the molecular basis for ATM function in protein homeostasis, the Paull lab identified a central role for PARP-dependent nuclear condensates arising from intrinsically disordered proteins associating with PARylated genomic sites (Lee et al., 2021). PARP activation in ATM deficient cells was shown to depend on increased oxidative stress, which in turn caused transcription-associated damage, RNA:DNA hybrid formation and ssDNA lesions. Of note, PARP activation was found to occur independently of oxidative lesions in ATLD patients, suggesting that alternative mechanisms exist to initiate PAR-dependent protein aggregation in ATLD. Of relevance for neurodegenerative disease, PAR-related protein-rich condensates were found to be wide-spread in A-T patient cerebellum (Lee et al., 2021). These findings point to an inherent danger of DNA damage-associated phase separation, particularly in the presence of excessive DNA damage or repair defects that may prevent its dynamic formation and resolution. It will be interesting to determine if other repair-defect- and/or RNA:DNA hybrid-associated neurodegenerative diseases exhibit similar pathology (Moreira et al., 2004; Loomis et al., 2014; Perego et al., 2019).

## Perspective

Three-dimensional nuclear organization is central to both accurate and aberrant genome maintenance. Continued efforts to map dynamic 3D changes in nuclear space in response to perturbations such as DNA damage, transcription or replication, are critical to advance our understanding of the pathways and factors that control genome maintenance. Recent advances in characterizing the mammalian nucleus in space and time, such as the NIH 4D Nucleome or the ENCODE projects (Consortium, 2004; Dekker et al., 2017), are providing relevant, high-resolution technologies and insight to inform future research in DNA repair. Some of the emerging issues in the field have been indicated throughout this review, but we would like to highlight a few key aspects we consider integral moving forward.

First, how does active movement of DSBs relate to the biophysical separation of repair environments via liquid demixing? Are these events part of the same process, or is there a choice between one and the other, and what would that choice depend on? Can we exploit aggregation and mobility mechanisms to manipulate repair processes, outcome and overall genome maintenance? The latter is supported by intriguing findings that inhibition of actin nucleation can sensitize cancer cells to both PARP inhibition and the DNA polymerase inhibitor aphidicolin (Schrank et al., 2018). Conversely, damage-induced liquid demixing appears to contribute to protein condensates associated with neurodegenerative disorders (Lee et al., 2021), while a potentially beneficial impact of phase separation on DNA repair reactions and concomitant genome maintenance remains to be identified.

Second, the aggregation of multiple DSBs within nuclear space, or at specialized micro-environments such as the NPC, begs the question of why distinct DSBs need to be brought together during repair. At first glance, this seems to be a dangerous proposition, as it may facilitate illegitimate repair events. Indeed, telomere fusions and dicentric chromosome formation are thought to be a result of this process (Lottersberger et al., 2015). What, then, are the benefits of specialized repair micro-environments? Or are DSB clusters merely a natural consequence of a condensation process that evolved to locally increase the concentration of repair factors? And what is the composition of phase separated condensates, HR-associated F-actin-dependent DSB aggregates and DNA lesion-associated NPCs? A better molecular and structural understanding of these specialized repair environments will no doubt help us determine their role in the repair process.

Third, although we may finally understand what leads to often Mb-sized DSB repair foci (Arnould et al., 2021), it remains a mystery as to why DSB marks such as γH2AX and its downstream effector proteins need to cover the extent of DNA they do. Placing these findings in the context of TADs will likely provide additional insight, but more work is needed to understand this most basic feature of DSB repair. Despite these remaining challenges, however, our understanding of DSB repair has advanced significantly with the consideration of a third nuclear dimension, as well as the complex arrangements of DNA lesions within nuclear space. We look forward to seeing this insight translated into actionable means to manipulate DNA repair to prevent or treat diseases associated with genome maintenance defects.
